# Supplementary material for: Sustainable Scalable Mechanochemical Synthesis of CdS/Bi2S3 Nanocomposites for Efficient Hydrogen Evolution
Source: Nanomaterials (Basel). 2024 Nov 6;14(22):1785. doi: 10.3390/nano14221785 (PMC11597724; doi:10.3390/nano14221785)
Supplement: Supplementary file 1 [file nanomaterials-14-01785-s001.zip › nanomaterials-3215828-supplementary.pdf]

Electronic Supplementary Information

The supporting information document contains 4 pages and 3 figures.

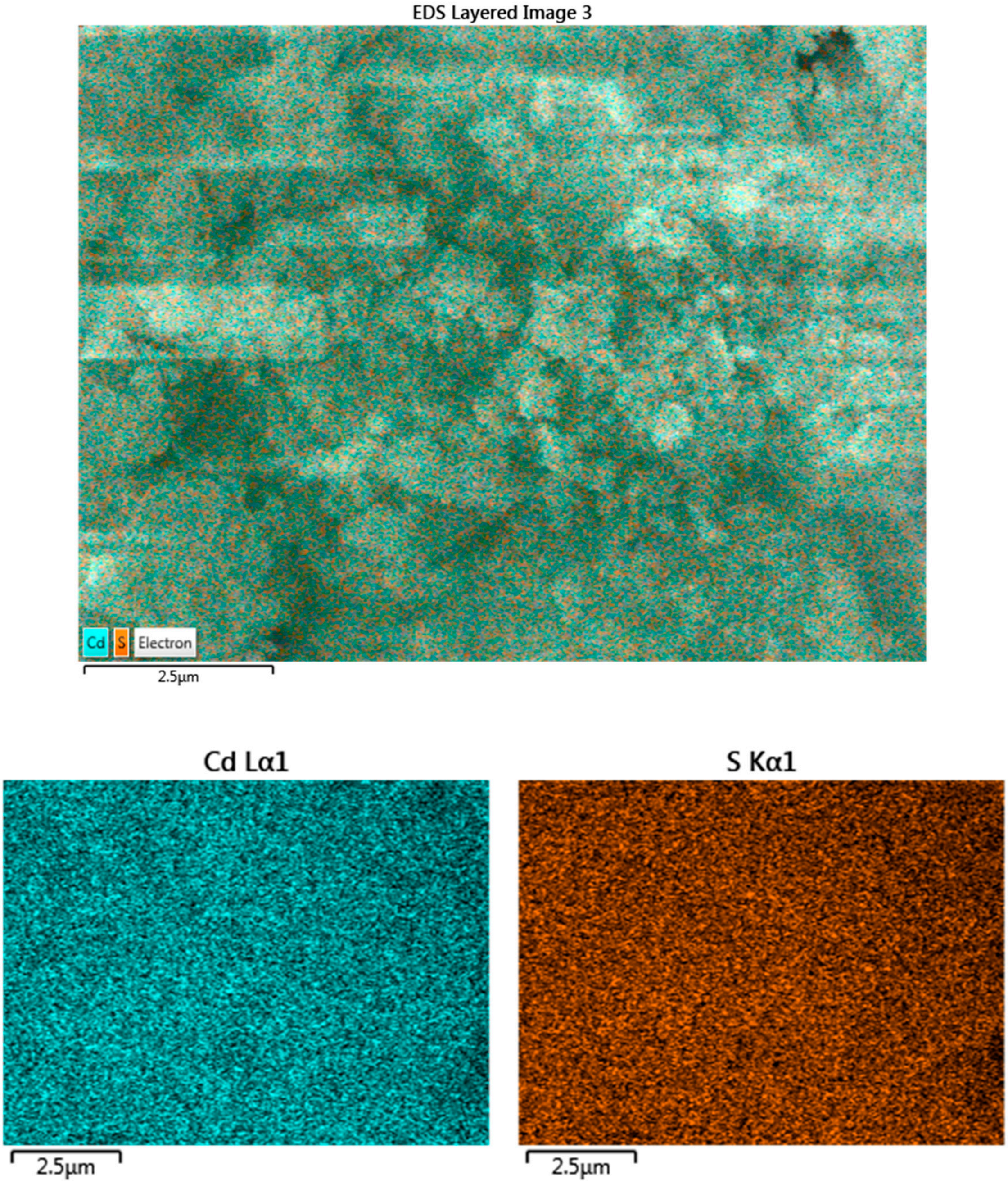

Figure S1. Elemental mapping of the CdS sample for Cd and S elements.

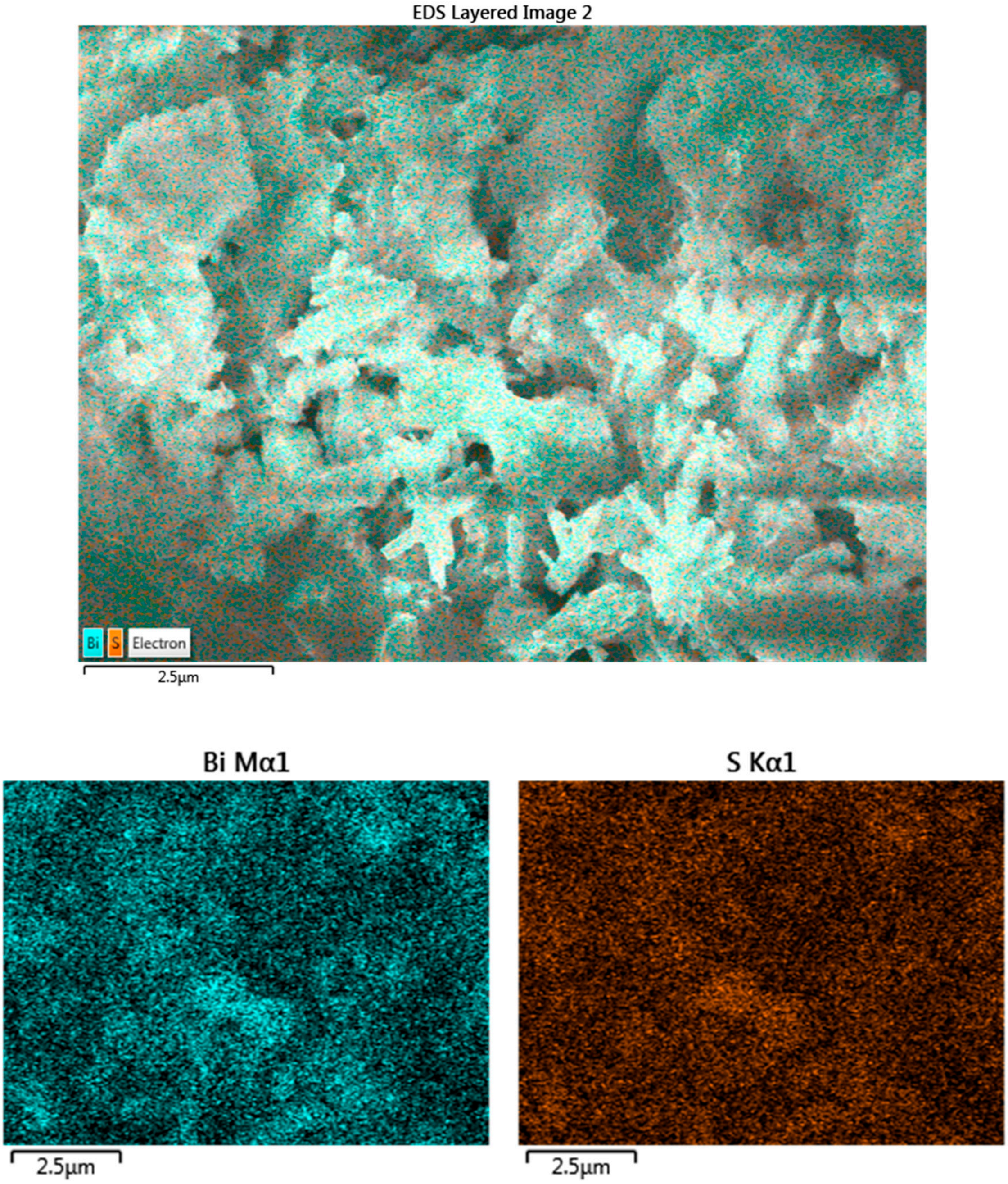

Figure S2. Elemental mapping of the Bi_2S_3 sample for Bi and S elements.

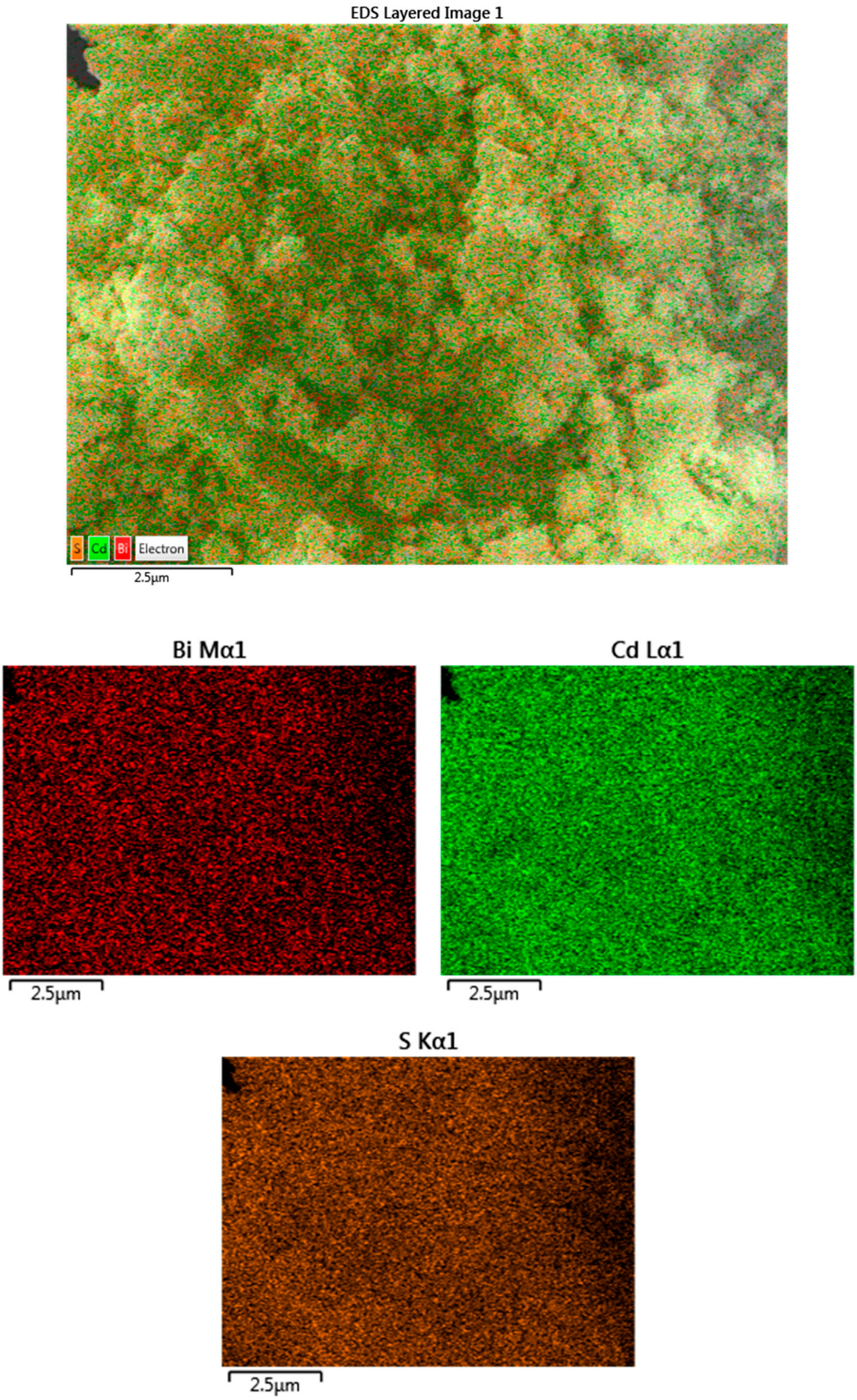

Figure S3. Elemental mapping of the CdS/Bi_2S_3 sample for Cd, Bi, and S elements.
